# Smoking and SARS-CoV-2: Are Polish health professionals at higher risk of infection?

**DOI:** 10.18332/tid/122760

**Published:** 2020-06-05

**Authors:** Paweł Koczkodaj, Magdalena Cedzyńska, Joanna Didkowska

**Affiliations:** 1 Cancer Epidemiology and Primary Prevention Department, Maria Sklodowska-Curie National Research Institute of Oncology, Warsaw, Poland

**Keywords:** Poland, smoking, health professionals, coronavirus, COVID-19

**Dear Editor,**

In Poland about 26% of adults smoke cigarettes, 31% of males (regular smokers 26%) and 21% of females (regular smokers 17%). The highest smoking prevalence is most pronounced among people aged 45–54 years (35%) and 55–64 years (32%), the lowest rates are observed among young adults aged 18–24 years. Moreover, within the last years, the decrease in smoking prevalence in Poland is due mainly to the lower number of smokers among males, currently about 9 percentage points (pp) less in comparison with 2012. In women, the drop was more modest at about 2 pp since 2012^1^.

**Figure 1:**
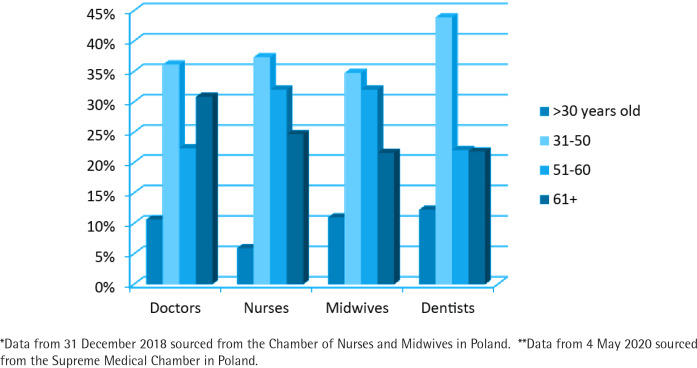
Percentage of nurses, midwives*, medical doctors and dentists** in Poland by age group

The smoking patterns may demonstrate an accelerating potential for further pandemic development in Poland. There is more and more evidence showing a significant correlation between smoking and higher risk of SARS-CoV-2 infection and COVID-19 development^2,3^. As mentioned, in Poland smoking prevalence is higher among the older age groups. Similarly, the risk of a severe course of COVID-19 increases with age^4^. These overlapping strong risk factors may be of crucial importance for the Polish general population, but notably for health professionals in Poland. The age structure of particular professional groups (Figure 1) is associated with higher risk of exposure in daily work as well as with potential smoking patterns that may result in drastic consequences for the healthcare system, where staff shortages are already severe.

Collected data show that the great majority of health professionals in Poland represent older age groups than younger ones, especially in the case of nurses and medical doctors. Smoking in this particular cohort can be considered as a potential threat for a further increase in the number of health professionals that are excluded from the healthcare system due to COVID-19 treatment and quarantine (in April 2020 there were about 4500 health professionals in quarantine^5^). Unfortunately, the data on smoking prevalence among Polish health professionals are very limited. There are a few studies presenting percentages of medical doctors, dentists, nurses and midwives who smoke (investigated groups were small and careful inference is recommended). For example, a study conducted in 2018^6^ (cohort of 423 physicians) showed that 7.8% of respondents were current smokers. Another study^7^ indicated that among 544 investigated dentists 13.2% were current smokers. On the other hand, Adamek et al.^8^ showed in their study that 40% of nurses smoked cigarettes.

Despite many limitations, the above data indicate that smoking is still a real health problem among health professionals, which nowadays has another important meaning. Even though the discussed data are very scarce, there is also other evidence showing that stress is a significant risk factor for smoking^9^ that may explain the higher smoking prevalence among health professionals.

In view of the above data and current epidemiological circumstances, we suggest to include recommendations on the importance of smoking cessation among health professionals to the national coronavirus medical guidance as well as to existing and future coronavirus public health campaigns.
